# Intrahepatic CD161^hi^CD8+T Cell Recruitment Has a Pathogenetic Potential in Chronic HBV Infection

**DOI:** 10.1002/iid3.70118

**Published:** 2025-01-12

**Authors:** Jianfei Li, Qian Liu, Wanlu Duan, Zhi Duan, Futing Liu, Mengqi Ruan, Qiyin Zong, Hao Zhang, Qiang Zhou, Qin Wang

**Affiliations:** ^1^ Department of Clinical Laboratory the Second Affiliated Hospital of Anhui Medical University Hefei China

**Keywords:** CD161^hi^CD8+T cells, chronic HBV infection, CXCL16, CXCR6

## Abstract

**Backgrounds and Aims:**

CD8+T cells are crucially associated with the fight against hepatitis B virus (HBV) infection. CD161 has been shown to express remarkably on HCV‐specific CD8+T cells. However, the accurate function of CD161+CD8+T cells in HBV immunity or pathogenesis remains undetermined.

**Methods:**

Blood samples were collected from 25 chronic hepatitis B (CHB) patients. Peripheral blood levels of CD161+CD8+T cells and their correlation with serum ALT levels were analyzed in CHB patients. To analyze the in vivo CD161+CD8+T cell's number, function, and intrahepatic recruitment characteristics, HBV replication mouse models were established. The expression of CD161 on HBV‐specific CD8+T cells was also detected by analyzing CD161+CD8+T cell functions during infection.

**Results:**

Patients with CHB infection had a markedly lower peripheral blood frequency of CD161+CD8+T cells than did healthy controls and negatively correlated with serum ALT level. Furthermore, compared to the control mice, the frequency of CD161+CD8+T cells was significantly decreased in the blood of acute and chronic HBV‐replicating mice. Moreover, CHB‐replicating mice had significantly increased hepatic levels of CD161+CD8+T cells, which was not observed in the acute group of mice. Additionally, the CD161+CD8+T cells were categorized into CD161^hi^ and CD161^int^CD8+T cells and it was revealed that in the liver of CHB‐replicating mice the primary recruited cells were CD161^hi^CD8+T. Intrahepatic CD161^hi^CD8+T cells demonstrated increased CXCR6 expression, enhanced production of cytokine IL‐17 and TNF‐ɑ, and reduced IFN‐γ secretion. Accordingly, the CXCL16 mRNA expression in the liver tissue of CHB‐replication mice was markedly higher than in acute HBV‐replicating and control mice. The study also revealed that HBV‐specific CD8+T cells were mainly CD161‐CD8+T cells.

**Conclusion:**

During HBV infection, the intrahepatic recruitment of CD161+CD8+T cells was mainly CD161^hi^CD8+T cell subpopulation, which has a weak antiviral response, but increased pro‐inflammatory effect, suggesting that CD161 may serve as a potential marker of liver‐damaging T cells.

## Graphical abstract

Peripheral blood CD161+CD8+T cells decrease and intrahepatic recruitment increases during chronic hepatitis B. The intrahepatic recruitment of CD161+CD8+T cells was mainly CD161^hi^CD8+T cell subpopulation. Intrahepatic recruitment of CD161^hi^CD8+T cells is associated with the CXCR6‐CXCL16 chemokine axis. CD161^hi^CD8+T cell frequency correlates with liver inflammation indicators. CD161hiCD8+T cells show stronger immunopathogenicity rather than promoting viral clearance

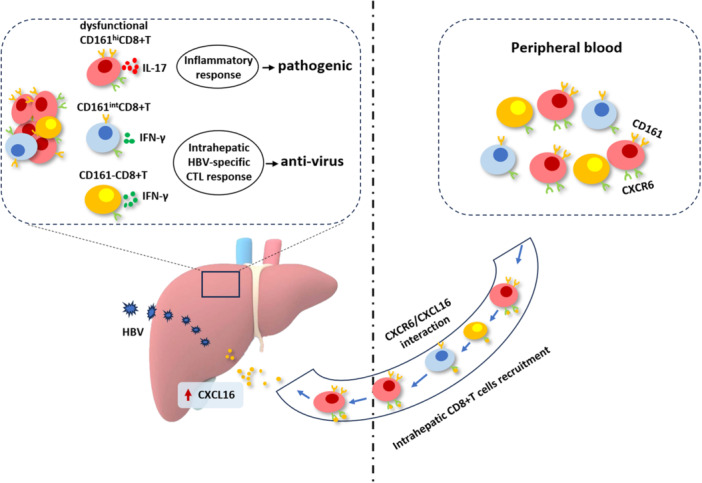

## 1 Introduction

Hepatitis B virus (HBV) affects the liver and may cause chronic hepatitis B (CHB) and progressively develop into cirrhosis and hepatocellular carcinoma in case the host's immune system fails to clear the virus [1]. Its clinical symptoms depend on a complex interaction between HBV replication and the host's immune reactions [2]. CD8+T cells are the primary effector cells responsible for virus clearance and disease pathogenesis, a fraction of antigen‐specific CD8+T cells clear viruses by killing the infected cells via cytolytic and non‐cytolytic mechanisms [3]. During infection, some CD8+T cells are recruited to the liver to exert inflammatory pathogenic effects, thus contributing to hepatic immunopathology [1, 4]. Originally discovered as a natural killer (NK) surface molecule (NK1.1), CD161 is also widely present in T cells and is associated with their tissue‐homing properties [5]. Furthermore, CD161 has been indicated to serve as a marker for MAIT, Tc17, and Th17 cells [6]. CD161 is exhibited on hepatitis B and C viruses but not on cytomegalovirus or influenza virus‐specific CD8+T cells. Their expression helps characterize functionally differentiated populations within a particular organ (e.g., the liver) that are responsive to viral infection [7]. The literature suggests that Tc cells with high CD161 expression are substantially abundant in the liver of CHB patients and express tissue‐homing‐associated chemokine receptors in the inflammatory state [5], suggesting that CD161+CD8+T cells might be recruited in the liver during HBV‐associated immunoinflammatory states. Another study reported that CD161+CD4+T cells aggregated in the HBV patient's liver and influenced the progression of liver fibrosis through the production of IFN‐γ and IL‐17 [8], whereas the effect that CD8+T cells expressing CD161 in the course of HBV infection is unclear. This research first identified that the levels of peripheral blood CD161+CD8+T cells were decreased and associated with hepatic inflammation in CHB patients. Therefore, it was hypothesized that these cell populations might converge from peripheral blood to the liver during HBV infection. Subsequently, HBV replicating mouse models were used to further explore the relevant features and functional states of CD161+CD8+T cells during HBV infection, and their association with viral clearance or immunopathogenesis.

## 2 Materials and Methods

### 2.1 Subject of the Study

#### 2.1.1 CHB Patients

Blood specimens from 25 CHB‐diagnosed patients who enrolled at the Second Affiliated Hospital of Anhui Medical University from September 2022 to November 2022 were collected. All patients met “The guideline of prevention and treatment for chronic hepatitis B (2019 version)” issued by the Chinese Society of Hepatology and Society of Infectious Diseases. Individuals with other non‐HBV hepatitis and other liver diseases were excluded. Furthermore, blood specimens from 15 healthy individuals were taken as healthy controls. The general data and some clinical indicators of the patients are shown in Table 1.

**Table 1:** General clinical indicators of patients.

Index	CHB	HC
	(*n* = 25)	(*n* = 15)
Age	39.4 ± 10.79	37.1 ± 9.64
Gender (male/female)	15/10	8/7
RBC (×10^12^/L)	4.6 ± 0.55	4.9 ± 0.46
WBC (×10^9^/L)	4.5 ± 2.01	6.4 ± 1.43
PLT (×9^12^/L)	162.5 ± 122.46	223.7 ± 37.14
Neut (%)	49.7 ± 9.87	58.1 ± 5.85
Lymph (%)	38.4 ± 9.01	31.9 ± 5.18
ALB (g/L)	43.9 ± 4.73	—
TBIL (μmol/L)	19.3 ± 17.04	—
ALT (U/L)	125.6 ± 187.10	—
AST (U/L)	85.5 ± 142.16	—
GGT (U/L)	64.6 ± 79.13	—

#### 2.1.2 Mice

Forty‐five healthy male 6‐week‐old C57BL/6 J mice, 20 g, SPF grade (Henan Skibbes Biotechnology Co. Ltd.; Anyang, China; license number: SCXK (YU)2020‐0005) were used for the establishment of the HBV replication model via hydrodynamic injection (HI) of HBV plasmids pSM2 or pAAV/HBV1.2. The injection comprised 10 μg of pSM2 or 6 μg of pAAV/HBV1.2 plasmids in saline solution with an equal volume of 0.1 mL/g of body mice weight and was injected into the tail vein in a relatively short period. As for the control group, an equal volume of saline was used in the same way as before.

### 2.2 Cell Isolation

Mouse intrahepatic lymphocytes (IHLs) were acquired by following the method described previously [9]. Immediately after euthanization, the mice's liver was perfused with PBS via the portal vein. Subsequently, the liver tissue was extracted, ground on a 70 μm filter to collect a hepatocyte suspension, centrifuged at 50 g for 5 min to separate the suspended hepatic nonparenchymal cells, resuspended in 40% Percoll, centrifuged again at 1000 g for 15 min. After removing the debris and hepatocytes in the upper layer, the IHLs in the precipitate were collected, washed, and analyzed. Mouse blood was obtained, and erythrocytes were lysed with erythrocyte lysis buffer (Servicebio, Wuhan, China) to prepare mouse peripheral blood single‐cell leukocyte suspensions.

### 2.3 Flow Cytometry

Cells were blocked with Fc blocker antibody and stained with the specific fluorescent monoclonal antibodies (mAbs), including APC‐A750 anti‐human CD8 mAbs, PE‐Cy7 anti‐human CD45 mAbs (Beckman Coulter, USA); FITC anti‐human CD64 mAbs, PE anti‐human CD161 mAbs, APC anti‐human CD3 mAbs (Uni‐medical, Shenzhen, China); FITC anti‐mouse CD4 mAbs, PerCP‐Cy5.5 anti‐mouse CD8 mAbs, PE‐Cy7 anti‐mouse CXCR6 mAbs, APC anti‐mouse CXCR3 mAbs, Pacific Blue anti‐mouse NK1.1 mAbs; FITC anti‐mouse TNF‐ɑ mAbs, PE anti‐mouse IL‐17A mAbs, PE‐Cy7 anti‐mouse CD4 mAbs, and APC anti‐mouse IFN‐γ mAbs (BioLegend, USA). For flow cytometry analysis, the surface and intracellular staining were performed as described previously [10]. To stain CD8+T cells specific to the K^b^‐HBV core 93–100 (MGLKFRQL), recombinant soluble dimeric mouse H‐2K [b]: Ig fusion protein (DimerX I, BD Bioscience, USA) were incubated overnight with HBV core 93–100 and then used to stain mouse lymphocytes, as per the manufacturer's guide. For intracellular cytokine staining, cells were stained with 5 μg/mL of CD3 and 1 μg/mL of CD28 at 37°C for 5 h in the presence of 3 μg/mL Brefeldin A (Invitrogen, USA). The cells were then fixed and permeabilized with intracellular fixation and permeabilization buffer set (Invitrogen, USA) and stained using FITC anti‐mouse TNF‐ɑ mAbs, PE anti‐mouse IL‐17A mAbs, and APC anti‐mouse IFN‐γ mAbs (BioLegend, USA). For analysis, BD FACS Lyric flow cytometer and FlowJo software were used. Dead cells and cellular debris are removed by fixable viability dye eFluor 780 and scatter signal.

### 2.4 qRT‐PCR

Total mouse liver RNA was extracted with Trizol (Shanghai Sangon Biotech, China), and cDNA was prepared using a reverse transcription kit (ToloBio, Shanghai, China). CXCL‐16 mRNA expression was detected using β‐Actin as an internal reference. The primers were synthesized by Shanghai Sangong Biotechnology, and the sequence (5′–3′) is F: CTCAGCACTCCACTCTTCCATCAG R: GTTTCTCATTTGCCTCAGCCTCAG(CXCL‐16); F: CTACCTCATGAAGATCCTGACC R: CACAGCTTCTCTTTGATGTCAC(β‐Actin). Reaction conditions were: pre‐denaturation at 95°C for 30 s, denaturation at 95°C for 10 s, annealing, and extension at 60°C for 30 s. The results were analyzed after running a total of 40 cycles.

### 2.5 Statistical Analysis

Graphing and statistical analysis were performed using GraphPad Prism 8 software and SPSS 27 software. The normally distributed data normality was assessed by the Shapiro–Wilk test. For intergroup differences comparison, a *t*‐test or one‐way ANOVA was performed and the correlation between the two groups was assessed by Pearson's correlation analysis. For non‐normally distributed data, intergroup differences were compared with non‐parametric tests, and Spearman's correlation analysis was employed to determine correlation. *p* < 0.05 was assumed to have statistical significance. *p < 0.05; **p < 0.01; ***p < 0.001.

## 3 Results

### 3.1 Decreased Frequency of Peripheral Blood CD161+CD8+T Cells in CHB Patients

**Figure 1:**
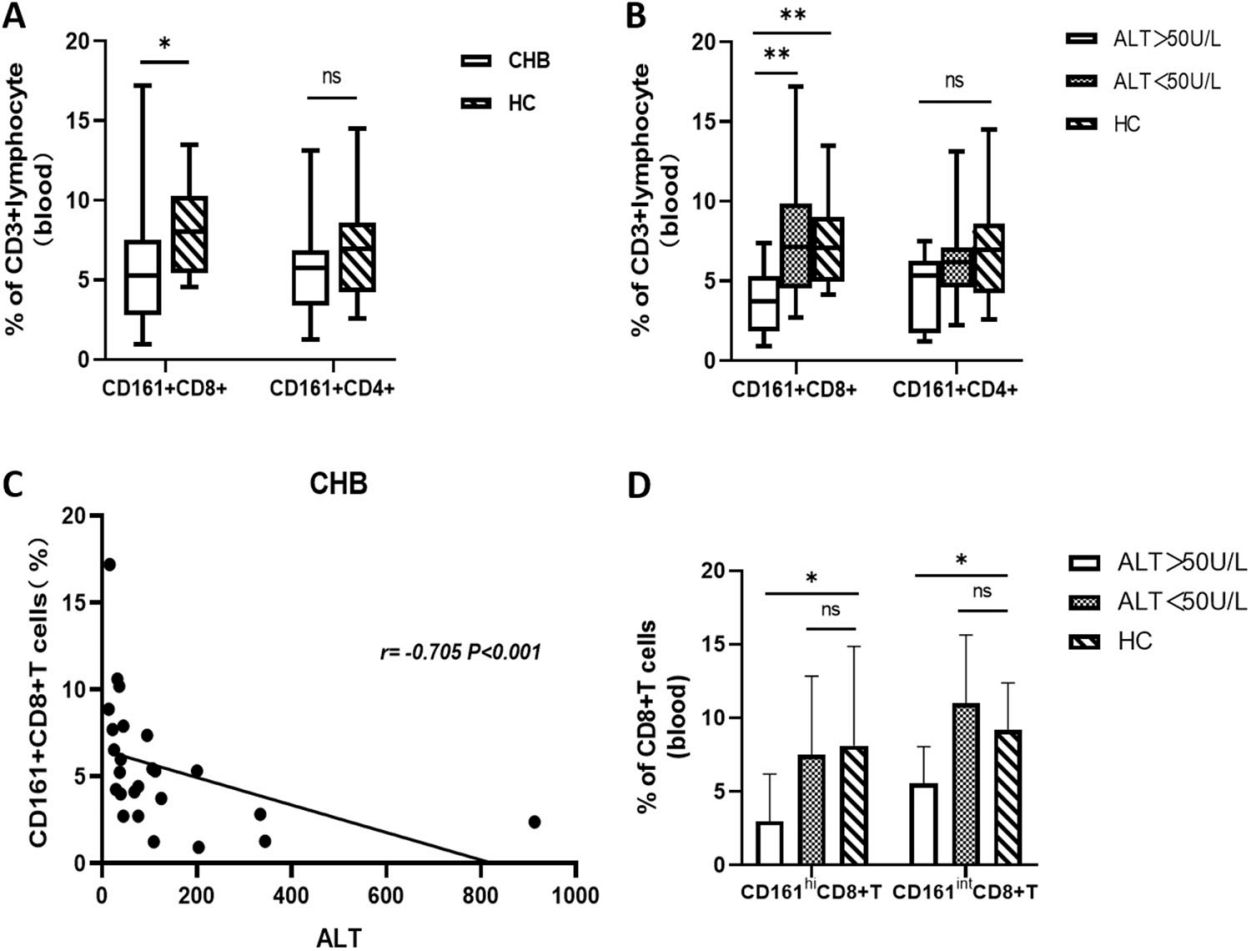
(A) Frequency of CD161+CD8+T and CD161+CD4+T cell in the peripheral blood of chronic hepatitis B patients. (B) Frequency of CD161+CD8+T and CD161+CD4+T cells in the peripheral blood of patients with different serum ALT levels; (C) Correlation between CD161+CD8+T cell frequencies in the peripheral blood of chronic hepatitis B patients and the serum ALT levels; (D) Frequencies of CD161^hi^CD8+T and CD161^int^CD8+T cells in the peripheral blood of chronic hepatitis B patients with different serum ALT levels. *p < 0.05, **p < 0.01.

The CD161 expression in peripheral blood T lymphocytes of CHB patients was analyzed. It revealed a significantly lower frequency of CD161+CD8+T cells in CHB patients than in healthy subjects, whereas no marked difference was observed in the frequency of CD161+CD4+T cells (Figure 1A). Furthermore, the patients were categorized into ALT‐elevated (*n* = 13) and ALT‐normal (*n* = 12) groups based on their ALT indexes (reference range = 9–50 IU/mL). It was revealed that the ALT‐elevated CHB group had a significantly lower frequency of CD161+CD8+T cells than those in the ALT‐normal group and healthy individuals, whereas no obvious distinction was observed between the ALT‐normal group and healthy individuals (Figure 1B). An obvious decrease of CD161^hi^CD8+T cells frequency in the peripheral blood of the ALT‐elevated CHB group was observed (Figure 1D). Moreover, there was no noticeable difference in the frequency of CD161+CD4+T cells among the three groups (Figure 1B). Correlation of CD161+CD8+T cell frequency with clinical indicators was detected and revealed a significant negative correlation with ALT, AST, and GGT, whereas no correlation was observed with HBsAg or HBV DNA (Figure 1C; Table 2).

**Table 2:** Correlation between CD161+CD8+T cells frequency and clinical indicators.

Index	CD161+CD8+T cells%	
	*r*	*p*
ALT	−0.705	< 0.001***
AST	−0.659	< 0.001***
GGT	−0.439	0.028*
ALB	0.056	0.791
TBIL	−0.386	0.057
HBsAg	−0.075	0.723
HBV DNA	−0.047	0.919
WBC	−0.073	0.736

* p < 0.05

*** p < 0.001.

### 3.2 Decreased CD161+CD8+T Cell Frequency in the Peripheral Blood and Enhanced Intrahepatic Infiltration of CD161+CD8+T Cells in Chronic HBV Replicating Mice

**Figure 2:**
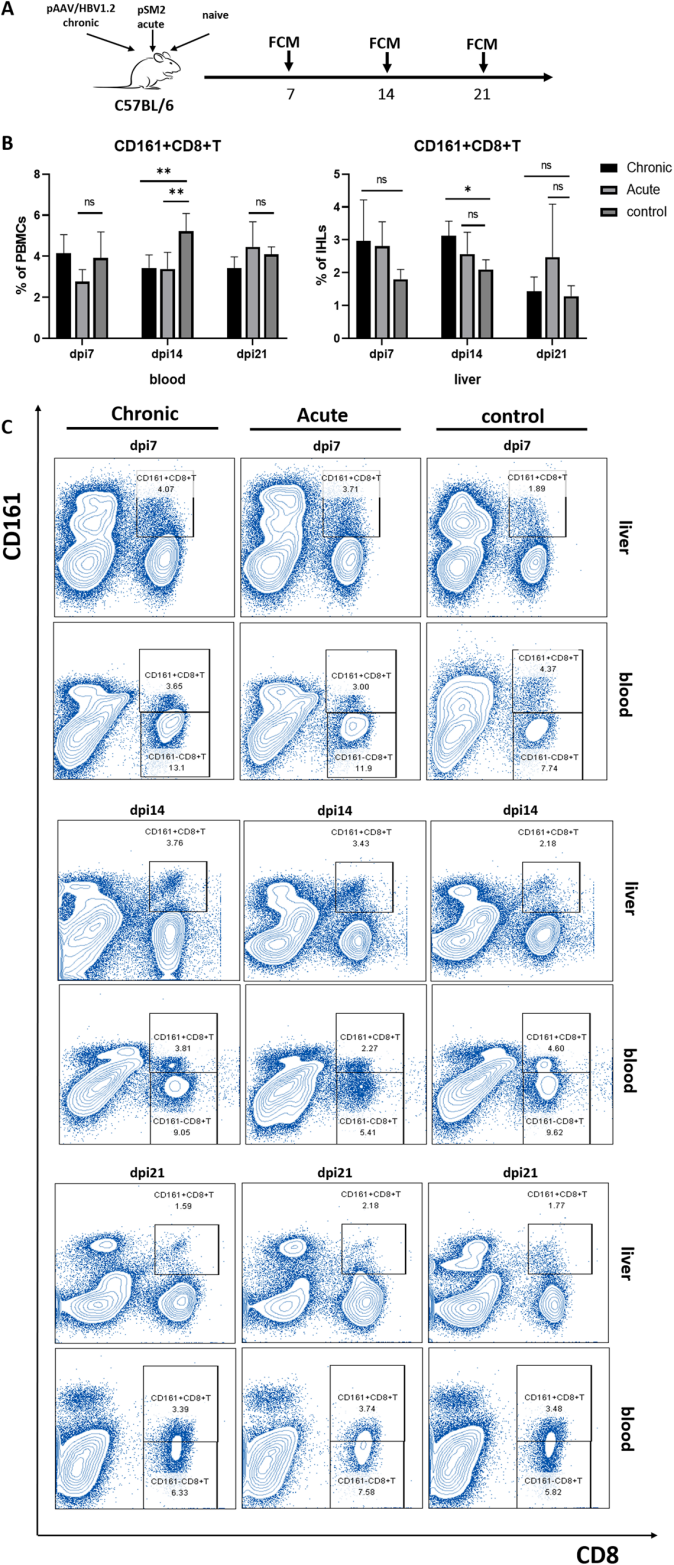
(A) Flow cytometry treatment of mice at the time points. (B) Flow cytometry detection of liver and peripheral blood CD161+CD8+T cell frequencies at different days after modeling of HBV replicating mice (*n* = 5). (C) Representative scatter plots. *p < 0.05, **p < 0.01.

To further explore the relevant properties of CD161+CD8+T cells during CHB infection, acute and chronic HBV replication models were constructed via HI of pSM2 and pAAV/HBV1.2 plasmids, respectively. Flow cytometric assays were performed at each of the three time points shown in Figure 2A. HI plasmid injection efficiently transferred the HBV genome into mouse hepatocytes, promoting the production of virus antigens, replication intermediates, and viral particles [11]. Injection of 10 μg pSM2 plasmid with HBsAg cleared at 14 days, whereas that of 6 μg pAAV/HBV1.2 plasmid indicated the sustainability of serum HBsAg positivity for > 4 weeks, suggesting a well‐characterized mouse model to emulate the acute and chronic HBV replication; its efficacy has been validated in previous studies [9]. The liver and peripheral blood CD161+CD8+T cells were analyzed in established HBV replication mouse models, which revealed that on Day 14 postinjection, peripheral blood CD161+CD8+T cell frequency was significantly lower in both acute and chronic groups compared to healthy controls. Furthermore, hepatic CD161+CD8+T cell frequency was elevated in the chronic group whereas the difference was not significant in the acute group (Figure 2B,C).

### 3.3 Increased CD161+CD8+T Cells in the Liver of Chronic HBV Replicating Mice Are Dominated by the CD161^hi^ Subpopulation

**Figure 3:**
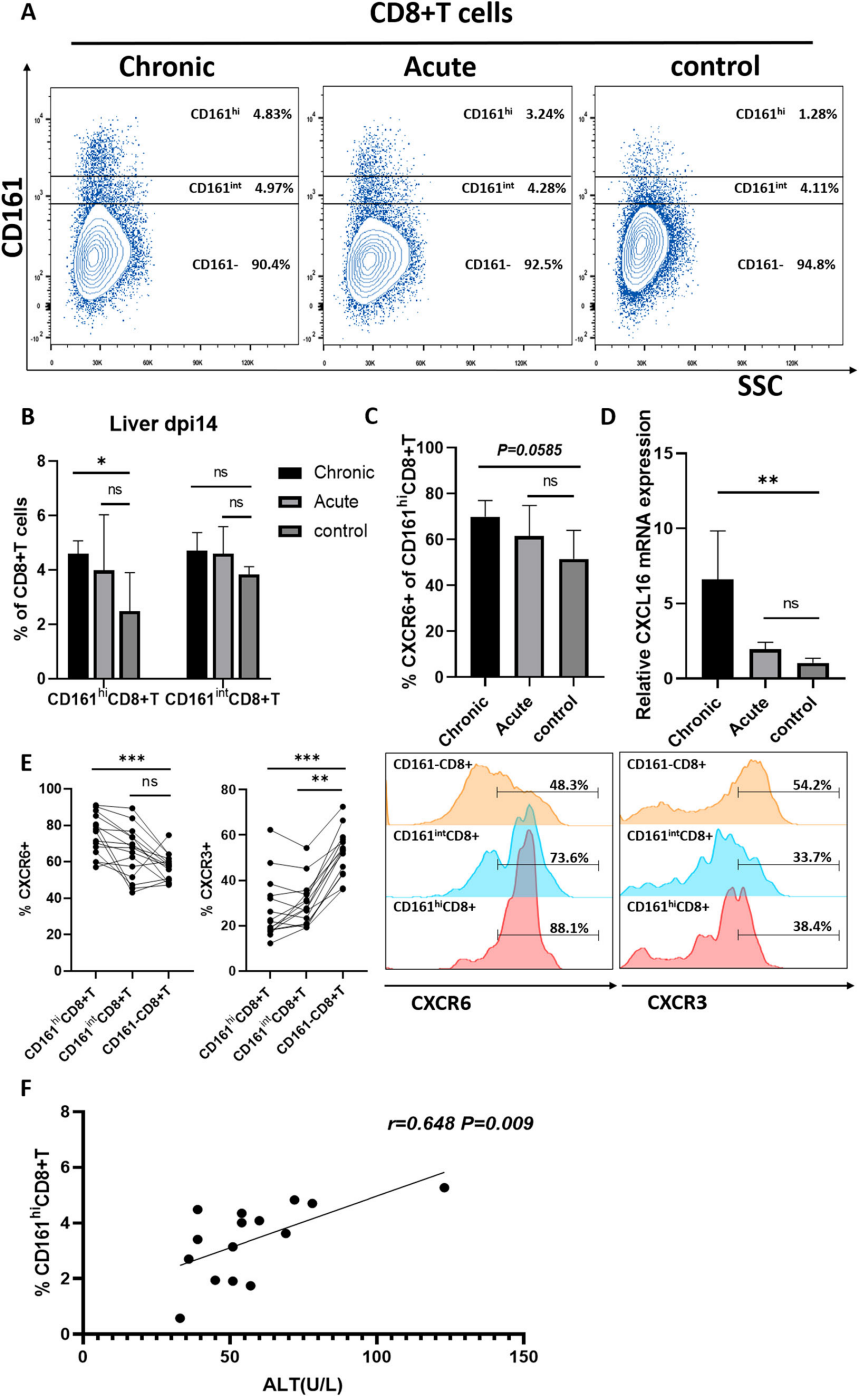
(A and B) Flow cytometry detection of intrahepatic CD161^hi^ and CD161^int^CD8+T cell frequencies in acute and chronic HBV replicating mice at Day 14 (*n* = 5). (C) Proportion of CXCR6+CD161^hi^CD8+T cells in acute and chronic HBV replicating mice at Day 14 (*n* = 5). (D) qRT‐PCR analysis of CXCL16 gene expression in the liver of chronic HBV replicating mice at Day 14 (*n* = 5). (E) Flow cytometry expression analysis of CXCR6 and CXCR3 on CD161^hi^ and CD161^int^CD8+T cell populations in the liver of chronic HBV replicating mice (*n* = 15). (F) Correlation between intrahepatic CD161^hi^CD8+T cell frequency and ALT index in chronic HBV replicating mice (*n* = 15). *p < 0.05, **p < 0.01, ***p < 0.001.

Two populations of CD161+CD8+T cells have been reported in the liver, differing in their CD161 expression, each of which has different functional properties [7]. The CD161+CD8+T cells were categorized into two groups according to the CD161 expression level (Figure 3A), that is, the CD161^hi^ clusters with high CD161 expression and the CD161^int^ clusters with intermediate CD161 expression. In the peripheral blood of patients with CHB, we found that the frequency of CD161^hi^CD8+T cells in the peripheral blood of CHB patients with elevated ALT levels was lower than that of patients with normal ALT levels or that of healthy individuals (Figure 1D); it was speculated that CD161^hi^CD8+T cell might be recruited into the liver leading to pathogenesis. In animal experiments, it was revealed that CD161+CD8+T cells were enhanced in the livers of mice in the chronic HBV replication group at 14 days. Subsequently, the frequency of their CD161^hi^CD8+T cells and CD161^int^CD8+T cells subpopulations was assessed, and it was indicated that the chronic group of mice had substantially increased frequency of CD161^hi^CD8+T cells in the liver than that in the control group, whereas no marked differences were observed for CD161^int^CD8+T cells, indicating that the increased CD161+CD8+T cells in liver of mice with chronic HBV replication are dominated the CD161^hi^CD8+T cells population. Increased CD161^hi^CD8+T cells were also observed in acute HBV‐replicating mouse livers, although no statistically significant difference was found (Figure 3B). Next, the expressions of the chemokine receptor CXCR6 on CD161^hi^CD8+T cells in the liver of the chronic mice group were categorized on Day 14. Compared with control mice, chronic HBV replicating mice exhibited a higher level of CXCR6 expression on CD161^hi^CD8+T cells, although it did not reach statistical significance (*p* = 0.0585) (Figure 3C). The intrahepatic CD8+T cells from 15 chronic HBV replicating mice that were processed at three different time points were analyzed. It was revealed that intrahepatic CD161^hi^CD8+T cells had elevated CXCR6 and reduced CXCR3 expression in comparison to CD161‐CD8+T cells. Moreover, CD161^int^CD8+T cells also displayed lower levels of CXCR3, but there was little difference in CXCR6 expression between them (Figure 3E). Additionally, the gene expression of intrahepatic chemokine CXCL16 was measured at 14 days, which revealed significantly increased expression in chronic HBV replicating mice than in the acute and control groups (Figure 3D). In comparison with the negative correlation between peripheral blood CD161+CD8+T cell frequency and its liver inflammation index in CHB patients, liver CD161^hi^CD8+T cell frequency in chronic HBV replicating mice showed a significant positive correlation with its ALT index (Figure 3F).

### 3.4 CD161^hi^CD8+T Cells Are High IL‐17‐Secreting, Low IFN‐γ‐Secreting Nonviral‐Specific Cells

**Figure 4:**
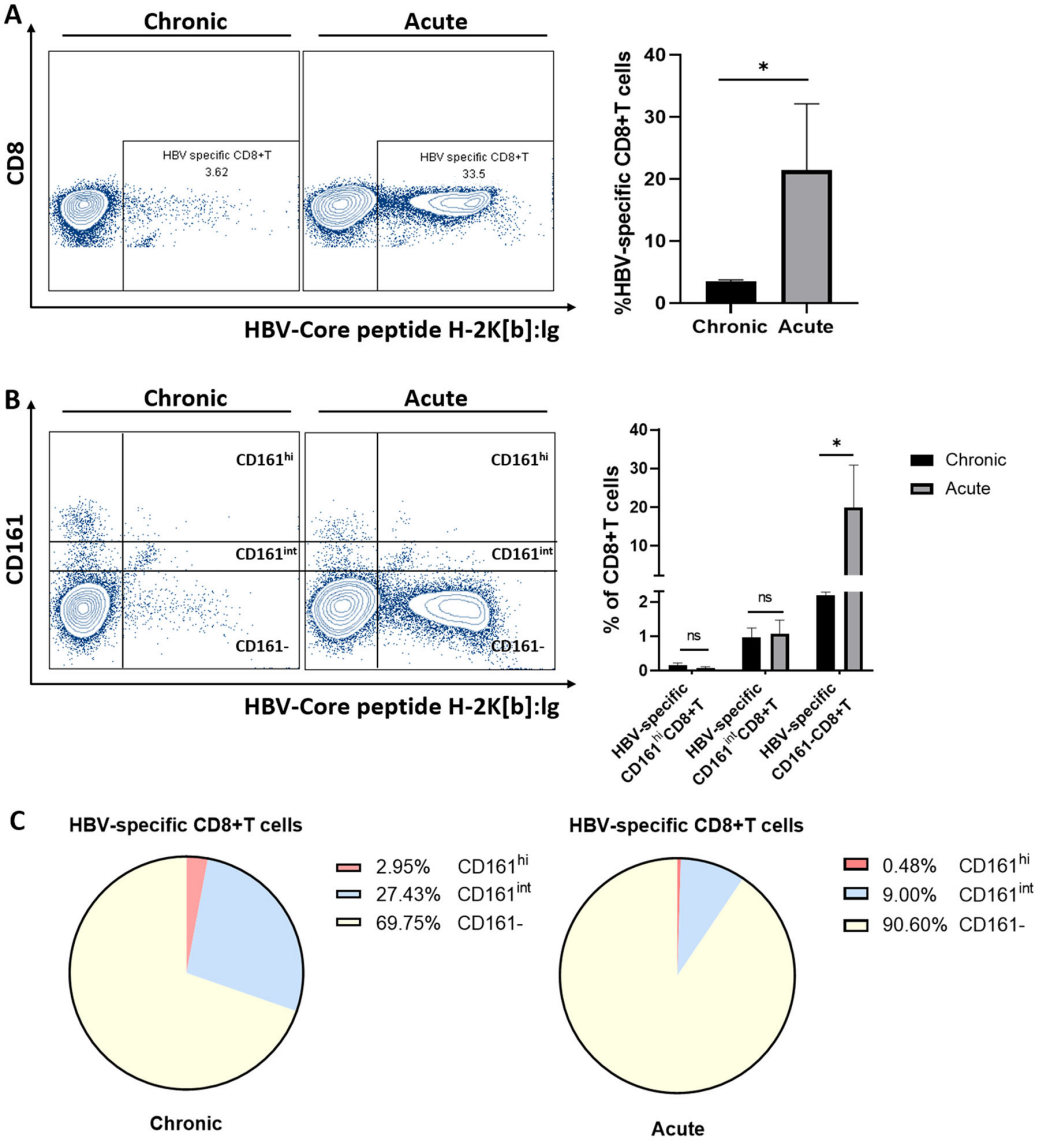
(A) Frequency of intrahepatic HBV‐specific‐CD8+T cells in HBV replicating mice at Day 21 as detected by flow cytometry (*n* = 4). (B) Frequency of intrahepatic CD161^hi^, CD161^int^, and CD161‐HBV‐specific‐CD8+T cells in HBV replicating mice at Day 21 (*n* = 4). (C) Map of the CD161 expression distribution in intrahepatic HBV‐specific‐CD8+T cells in HBV replicating mice at Day 21 (*n* = 4). *p < 0.05.

**Figure 5:**
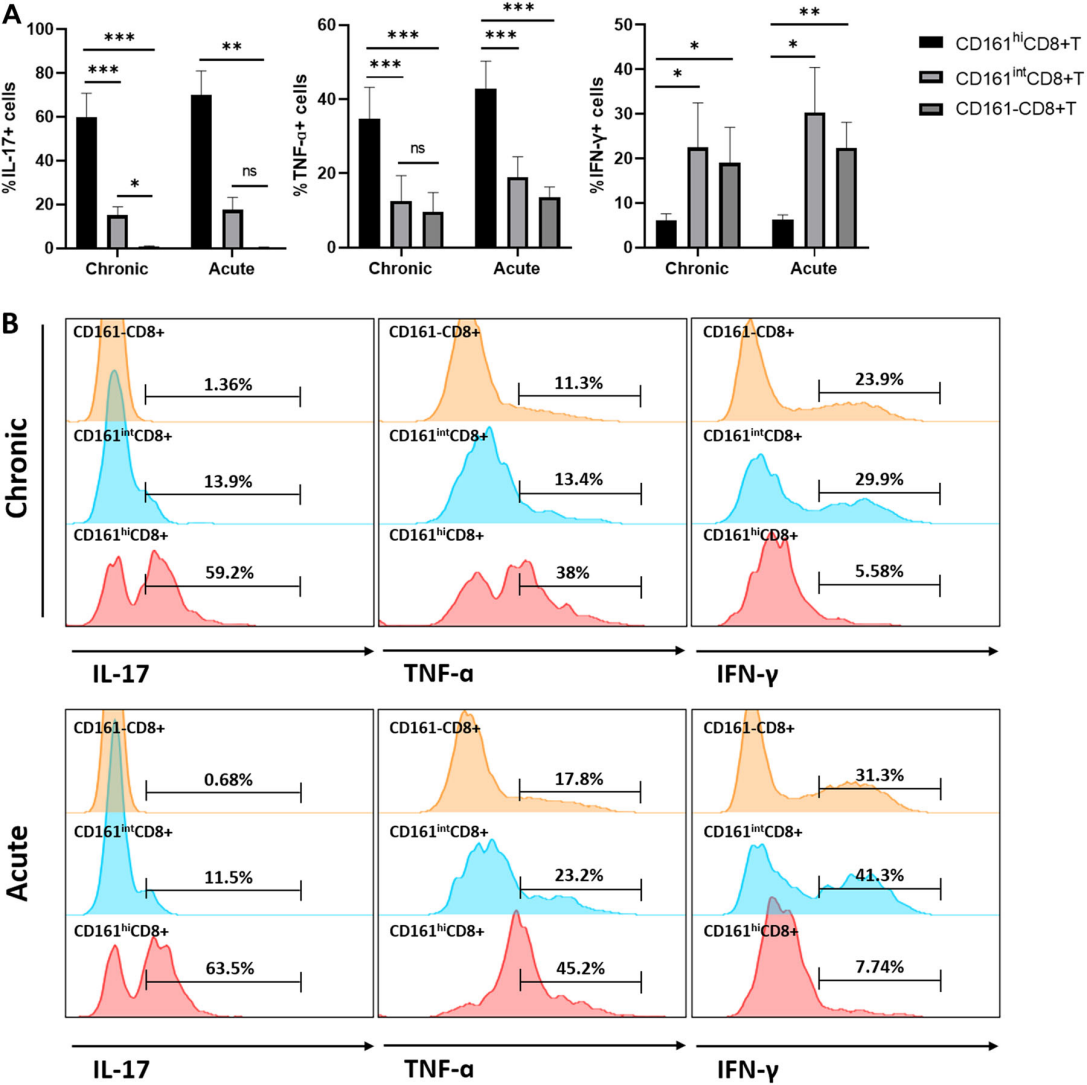
(A and B) Intrahepatic lymphocytes from acute and chronic HBV replicating mice were subjected to short‐term stimulation with CD3+CD28 at Day 14 before flow cytometry analysis to detect IL‐17, TNF‐ɑ, and IFN‐γ producing capacity of CD161^hi^, CD161^int^, and CD161‐CD8+T cells (*n* = 5). *p < 0.05, **p < 0.01, ***p < 0.001.

HBV‐specific CD8+T cells were assayed in acute and chronic HBV‐replicating mouse livers using Dimer staining. As Figure 4A depicts, it was observed that there were significantly more HBV‐specific CD8+T cells in the liver of acute HBV‐replicating mice than in the chronic group. Further analysis of the expression of CD161 on HBV‐specific CD8+T cells revealed that the multiplicity of HBV‐specific CD8+T cells of the acute HBV replicating mice was mainly the CD161 population. Furthermore, the frequency of HBV‐specific CD161^hi^CD8+T and CD161^int^CD8+T cells did not differ between the acute and chronic groups, and their hepatic frequency was low (Figure 4B). In contrast to a proportion of HBV‐specific CD8+T cells expressing intermediate and high levels of CD161, HBV‐specific CD8+T cells are dominated by the CD161‐cell cluster (Figure 4C). IHLs were taken from acute and chronic HBV replicating mice at Day 14 and were stimulated for a short time by CD3+CD28 in vitro; it was shown that after stimulation CD161^hi^CD8+T cells displayed stronger IL‐17 and TNF‐ɑ secretion compared to CD161^int^CD8+T and CD161‐CD8+T cells, but their IFN‐γ secretion capacity was weaker (Figure 5A,B).

**Figure 6:**
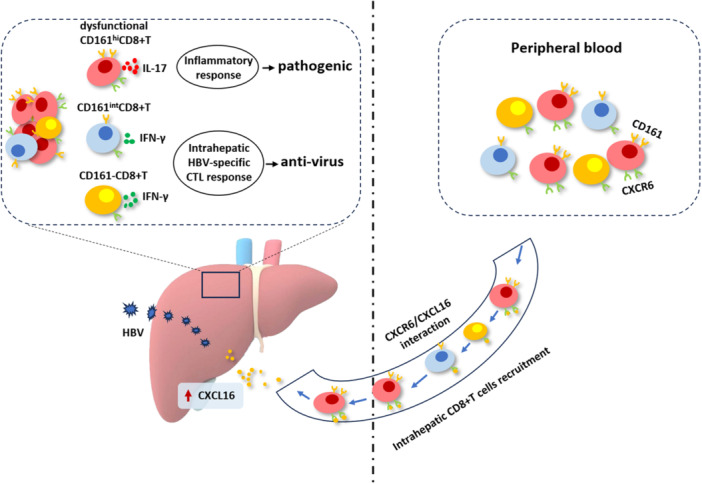
Graphical abstract of the function of CD8+T cells with different expressions of CD161 during HBV infection.

Overall, our study demonstrates that CD161^hi^CD8+T cells are recruited to the liver during chronic HBV infection via CXCR6‐CXCL16 interaction. Hepatic CD161^hi^CD8+T cells show weak antiviral function but increased pro‐inflammatory response, indicating the pathogenic potential of CD161^hi^CD8+T cells in chronic HBV infection (Figure 6).

## 4 Discussion

CD161 was originally identified as the mouse NK cell surface marker, NK1.1, expressed in NK and T cells, NKT cells, monocytes, and dendritic cells [12]. The distribution of CD161+T cells marked tissue‐organ variability, accounting for 1%–2% of all T cells in the thymus, blood, and secondary lymphoid tissues, whereas 20%–40% in the liver [13]. CD161 expression on T cells may mark its unique tissue‐homing properties. In hepatitis C patients' livers, CD161+CD8+T cells are highly enriched and express significantly higher liver‐associated chemokine receptors. Li et al. reported that CD161+CD4+T cells accumulate in the liver during CHB and are associated with the progression of liver fibrosis [5, 8]. This study noticed that CD161+CD8+T cells were lost in the peripheral blood of CHB patients, and the frequencies were negatively correlated with liver inflammation indicators, including ALT, AST, and GGT. It was hypothesized that the decreased population of cells in the blood may be because of their recruitment to the liver. Therefore, a simulated HBV replicating mouse model was established with plasmids, analyzed, and revealed a significant rise in the frequency of intrahepatic CD161+CD8+T cells and a significant decrease in their frequency in peripheral blood on Day 14 after chronic HBV modeling, suggesting that they are recruited to the liver during CHB. However, on Day 21 after the construction of the chronic model, a similar increase in the frequency of intrahepatic CD161+CD8+T cells was not observed on Day 14, instead, they showed a reduction.

CD161+T cells showed a variable degree of NK cell‐like innate activity, which in some cases is considered to be a marker of T cell “innate” [14]. In humans, CD8+T cell subpopulations with intermediate and high expression of CD161 exist, CD161^hi^ exhibit a Th17‐related differentiation phenotype, up‐regulate the expression of RORγt, CCR6, IL18R, and are the predominant type of IL‐17‐producing CD8+T cells; therefore, they were described as Tc17 cells, whereas, CD161^int^ are a specialized population of memory CD8+T cells with effector functions [5, 15]. Tc17 is widely implicated in immunological processes in various diseases, such as gastrointestinal‐related cancers. The intratumor Tc17 cell count increases with tumor progression and is associated with a poorer patient survival rate [16]. Additionally, Tc17 cells have also been shown to be enriched in CNS tissues in multiple sclerosis [17]. Here, it was identified that CD161^hi^CD8+T cells as the major population among the increased CD161+CD8+T cells recruited within the liver of CHB patients. The aggregated CD161^hi^CD8+T in hepatitis C livers highly expresses CXCR6 [5], consistent with this study. CXCR6 functions as an essential liver‐associated tissue homing‐related chemokine receptor that binds to the chemokine CXCL16 [18], which is widely expressed in the liver, lung, and other organs, to mediate the directed cellular chemotaxis. Furthermore, this research confirmed that CD161^hi^CD8+T cells with increased CXCR6 expression were higher in frequency in the liver of chronic HBV replicating mice than that of the control group. Moreover, it was discovered that the expression of the CXCL16 gene was increased in the liver of the chronic HBV replicating mice, while the acute HBV replicating mice did not exhibit elevated expression, explaining why the increase of CD161^hi^CD8+T cells in the liver of the chronic group of mice is not significant in the acute group. CD161^hi^CD8+T cells were recruited to the liver to exert their immune effects by upregulating CXCR6 expression, mediated by the intrahepatic CXCL16 chemokine. CXCR3 is also an important lymphocyte chemotactic receptor and is highly expressed on the surface of CD161+CD4+T cells in CHB patients' liver [8]. However, it was found that its expression on CD161^hi^CD8+T cells was lower than that on CD161‐CD8+T cells; the reason for this difference might be attributed to the different compositions of the Tc17 cell subpopulations. CXCR3+CCR4‐cells were characterized as Tc17.1 cells, expressing IL‐17 and IFN‐γ, whereas CXCR3‐CCR4+cells were Tc17 cells, which highly express IL‐17 but not IFN‐γ [19]. It was, therefore, hypothesized that increased CD161^hi^CD8+T cells in the liver might be biased toward Tc17 cells that do not express CXCR3 rather than Tc17.1, which is consistent with our observation that CD161^hi^CD8+T cells hyper‐secrete IL‐17 but barely secrete IFN‐γ after in vitro CD3+CD28 stimulation.

CD8+T cells as critical effector cells for HBV clearance by inducing interferon‐γ secretion, which mediates the non‐cytolytic mechanism that plays a crucial function in the viral clearance [3, 20]. HBV‐specific CD8+T cells are usually deficient in CHB patients [21], consistent with in vivo mouse models in this study. It was identified that CD161^hi^CD8+T cells did not exhibit viral specificity, 0.21% and 4.19% of HBV‐specific CD8+T cells express high and intermediate levels of CD161, respectively. CD161^hi^CD8+T cells are a population of non‐virus‐specific cells, while their low IFN‐γ secretion capacity indicates that they don't have a primary role in HBV clearance (Figure 4B). Although it was observed that CD161^int^CD8+T cells contained a percentage of HBV‐specific cells, similar to previous studies [7], an increase in the frequency of HBV‐specific CD161^int^CD8+T cells was not observed in the acute HBV replication model, and their role in the HBV clearance process needs further confirmation. Recent studies have reported that CD161 is a glioblastoma inhibitory immune checkpoint and blocking CD161 receptors on tumor‐infiltrating T cells enhances their immune effects and tumor‐killing potency in vivo and in vitro [22]. Furthermore, IL‐17 can directly promote immunosuppression and inhibit interferon release and proliferation of CD8+T cells by mediating infiltration of myeloid‐derived suppressor cells (MDSCs) via CXCL12 [16]. It has been suggested that the aggregation of CD161^hi^CD8+T cells within the CHB patient's liver is potentially detrimental to viral clearance. CD161 is the unique marker for almost all IL‐17‐secreting lymphocytes in the body, including Tc17 and Th17 [23]. This study indicated that only the CD161^hi^CD8+T cell subset secreted significant amounts of IL‐17, whereas CD161^int^CD8+T and CD161‐CD8+T cells secreted less and almost no IL‐17. Moreover, IL‐17 has strong immunopathogenicity and can promote the generation of pro‐inflammatory factors by a variety of inflammatory cells, such as macrophages, and exacerbate the progression of inflammation [24]. IL‐17 also amplifies and cooperates with IL‐8, which diminishes the antiviral effects of IFN‐γ and recruits neutrophils to mediate increased inflammatory injury [25, 26, 27]. Some studies have observed significantly enhanced IL‐17 expression in CHB liver tissues, especially in liver fibrosis. It closely correlates with the extent of liver fibrosis [28]. This research indicated a significant positive correlation between intrahepatic CD161^hi^CD8+T cell frequency and ALT, an indicator of inflammation, in CHB mice. Infiltration of CD161^hi^CD8+T cells secreting high IL‐17 levels in the liver may contribute to the progression of intrahepatic inflammation and exacerbate hepatic injury, thereby causing severe hepatic injury, which could facilitate further recruitment of CD161^hi^CD8+T cells, ultimately leading to cell exhaustion. Therefore, the high expression of CD161 on CD8+T cells probably represents a population of cells with high inflammatory effects that might be potential targets for hepato‐protective therapy for HBV‐infected patients with liver injury.

## 5 Conclusion

In summary, CD161^hi^CD8+T cells are a class of Tc17‐like cells and during HBV infection, they have a weaker antiviral effect, causing a stronger pro‐inflammatory hepatocellular injury effect. Furthermore, these cells participate in the development of chronic HBV inflammation. However, whether the infiltration of CD161^hi^CD8+T cells suppresses antiviral immunity and causes a delay in HBV clearance is unclear and requires further studies.

## Author Contributions

**Qiang Zhou:** conceptualization, methodology, project administration. **Qin Wang:** conceptualization, methodology, resources, formal analysis, writing–review and editing, funding acquisition. **Jianfei Li:** data curation, formal analysis, investigation, writing–original draft, visualization. **Qian Liu:** data curation, formal analysis, investigation. **Wanlu Duan:** software, supervision. **Zhi Duan:** investigation. **Futing Liu:** investigation. **Mengqi Ruan:** investigation. **Qiyin Zong:** investigation. **Hao Zhang:** validation, supervision.

## Ethics Statement

The research involving human participants was approved by the Ethics Committee of the Second Hospital of Anhui Medical University (20190065). Participants' legal guardians/next of kin provided written informed consent to participate in this study. All animal experiments were reviewed and approved by the Institutional Animal Care and Use Committee of Anhui Medical University (20190091).

## Conflicts of Interest

The authors declare no conflicts of interest.

## Data Availability

The raw data supporting the conclusions of this article will be made available by the authors without reservation.
